# On Top of the Alveolar Epithelium: Surfactant and the Glycocalyx

**DOI:** 10.3390/ijms21093075

**Published:** 2020-04-27

**Authors:** Matthias Ochs, Jan Hegermann, Elena Lopez-Rodriguez, Sara Timm, Geraldine Nouailles, Jasmin Matuszak, Szandor Simmons, Martin Witzenrath, Wolfgang M. Kuebler

**Affiliations:** 1 Institute of Functional Anatomy, Charité-Universitätsmedizin Berlin, 10117 Berlin, Germany; lopez-rodriguez.elena@charite.de; 2 German Center for Lung Research (DZL), 10117 Berlin, Germany; martin.witzenrath@charite.de (M.W.); wolfgang.kuebler@charite.de (W.M.K.); 3 Research Core Unit Electron Microscopy and Institute of Functional and Applied Anatomy, Hannover Medical School, 30625 Hannover, Germany; hegermann.jan@mh-hannover.de; 4 Core Facility Electron Microscopy, Charité-Universitätsmedizin Berlin, 10117 Berlin, Germany; sara.timm@charite.de; 5 Department of Infectious Diseases and Respiratory Medicine, and Division of Pulmonary Inflammation, Charité-Universitätsmedizin Berlin, 10117 Berlin, Germany; nouailles.geraldine@charite.de; 6 Institute of Physiology, Charité-Universitätsmedizin Berlin, 10117 Berlin, Germany; matuszak.jasmin@charite.de (J.M.); szandor.simmons@charite.de (S.S.)

**Keywords:** lung, alveoli, air-blood barrier, epithelium, air-liquid interface, alveolar lining layer, glycocalyx, surfactant

## Abstract

Gas exchange in the lung takes place via the air-blood barrier in the septal walls of alveoli. The tissue elements that oxygen molecules have to cross are the alveolar epithelium, the interstitium and the capillary endothelium. The epithelium that lines the alveolar surface is covered by a thin and continuous liquid lining layer. Pulmonary surfactant acts at this air-liquid interface. By virtue of its biophysical and immunomodulatory functions, surfactant keeps alveoli open, dry and clean. What needs to be added to this picture is the glycocalyx of the alveolar epithelium. Here, we briefly review what is known about this glycocalyx and how it can be visualized using electron microscopy. The application of colloidal thorium dioxide as a staining agent reveals differences in the staining pattern between type I and type II alveolar epithelial cells and shows close associations of the glycocalyx with intraalveolar surfactant subtypes such as tubular myelin. These morphological findings indicate that specific spatial interactions between components of the surfactant system and those of the alveolar epithelial glycocalyx exist which may contribute to the maintenance of alveolar homeostasis, in particular to alveolar micromechanics, to the functional integrity of the air-blood barrier, to the regulation of the thickness and viscosity of the alveolar lining layer, and to the defence against inhaled pathogens. Exploring the alveolar epithelial glycocalyx in conjunction with the surfactant system opens novel physiological perspectives of potential clinical relevance for future research.

## 1. The Alveolar Epithelium of the Lung and its Surfactant Lining

The architecture of the lung is optimized to serve its main function, gas exchange. A large surface area for air and blood (about 120 m^2^) with minimal distance (about 2 µm) is distributed over hundreds of millions of alveoli. The wall separating neighbouring alveoli contains three tissue compartments that constitute the air-blood barrier: alveolar epithelium, capillary endothelium, and the interstitium in-between. The basic knowledge about the structure and function of the alveolar epithelium seems to be well established. It is a continuous layer constituted by a mosaic of two cell types with specific differentiation. While alveolar epithelial type I (AEI) cells are specialised lining cells, alveolar epithelial type II (AEII) cells are specialized secretory and progenitor cells. AEI cells, which are less frequent than AEII cells, cover the vast majority of the alveolar surface with their branched thin squamous cell extensions [1]. Interspersed are single cuboidal AEII cells, which are easily recognized by their characteristic secretory organelles, the surfactant-storing lamellar bodies. Epithelial renewal and repair of both cell types is provided by AEII cells (for review, see [2,3]).

Soon after the first demonstration of a continuous alveolar epithelium in the mammalian lung by electron microscopy (EM) [4,5], it became obvious that this epithelium is not naked. It is covered by a fluid alveolar lining layer consisting of two phases: a surface film and an aqueous hypophase [6,7]. Later cryo-EM studies have confirmed the existence of a thin and continuous alveolar lining layer [8]. Surfactant, the secretory product of AEII cells, is a central component of this layer, i.e., it exerts its functions at the air-liquid interface of lung alveoli (for review, see [2,9,10,11,12,13]).

Surfactant is complex, both biochemically and ultrastructurally. It consists of about 90% lipids (mainly saturated phospholipids) and about 10% proteins (including the surfactant proteins SP-A, SP-B, SP-C and SP-D). All surfactant components are synthesized, stored, secreted and to a large extent recycled by AEII cells. Intracellular surfactant (at least lipids and the hydrophobic SP-B and SP-C) is assembled in lamellar bodies prior to secretion. Intraalveolar surfactant includes the surface film and morphologically distinct subtypes in the hypophase. These subtypes largely correspond to different stages in surfactant metabolism and activity. According to current models, freshly secreted surface-active lamellar body material transforms into tubular myelin, which is a potential precursor of the surface film. Additional multilayered surface-associated surfactant reservoirs have also been suggested. Inactive surfactant is usually present as small unilamellar vesicles. These can either be taken up by AEII cells for recycling or degradation or taken up by alveolar macrophages for degradation. Thus, the hypophase of the alveolar lining layer provides both a substrate on which surfactant acts as well as a reservoir in which intermediates of surfactant metabolism (precursors and remnants) are located.

The functions of surfactant are biophysical as well as immunomodulatory, with its individual components contributing to these functions in different and specific ways. The hydrophobic SP-B and SP-C are assigned mainly biophysical relevance whereas the hydrophilic SP-A and SP-D, which belong to the protein family of collectins, are considered to serve mainly immunomodulatory functions. Regarding its biophysical functions, surfactant stabilizes alveolar dimensions and thus prevents alveolar collapse by a surface-area dependent reduction of alveolar surface tension. Moreover, the low surface tension at the alveolar air-liquid interface secured by surfactant also prevents intraalveolar edema formation. Surfactant is therefore essential for normal alveolar micromechanics and lung function by keeping alveoli homogeneously open, dry and clean (reviewed in [2,11,13]).

## 2. The Glycocalyx of the Alveolar Epithelium

The Greek term “glycocalyx” can be translated as “sweet husk” [14]. It is a sugar-rich external cell coat common in many cell types, in particular where facing a lumen. The glycocalyx is anchored to the apical cell membrane and consists of two main backbone molecule classes: proteoglycans with long unbranched glycosaminoglycan side chains and glycoproteins with short branched carbohydrate side chains. Proteoglycan core proteins can be attached to the cell membrane by a transmembrane domain (e.g., syndecans) or by a glycosylphosphatidylinositol anchor (e.g., glypicans). Glycosaminoglycans are long negatively charged polymers consisting of disaccharide subunits. Common glycosaminoglycans in the glycocalyx are heparan sulphate, chondroitin sulphate and the un-sulphated hyaluronan. Hyaluronan differs from the other glycosaminoglycans in that it is (at least in part) not directly bound to a core protein but either binds to its receptor CD44 or intercalates throughout the glycocalyx, thereby contributing to hydration and viscosity. Other molecules and ions are attached to this cell surface coat, thus forming a highly hydrated dynamic meshwork that can function as a regulator for barrier permeability by maintaining osmotic gradients, as a regulator for cell adhesion or as a mechanosensor (reviewed in [15,16,17]).

The pulmonary endothelial glycocalyx recently gained attention because of its role in various lung diseases such as acute respiratory distress syndrome and pulmonary arterial hypertension [17,18,19,20]. However, much less is known about the glycocalyx of the alveolar epithelium. Compared to the available literature on surfactant, it seems that it has almost been overlooked as a component of the epithelial lining in lung alveoli. Only few studies have focussed on or at least appreciated the existence of an alveolar epithelial glycocalyx. One finds some early EM studies which reported the visualization of an alveolar epithelial glycocalyx (see below). This line of research was not pursued further until recently. Based on previous reports on alveolar shedding of syndecans in models of lung injury [21,22], Haeger et al. reported the presence of heparan sulphate at the alveolar surface and its shedding into alveolar spaces, together with syndecans 1 and 4, in mice subjected to lipopolysaccharide-induced acute lung injury. This led to increased lung protein permeability and thus a loss of barrier function. The shedding of heparan sulphate was at least partly mediated by matrix metalloproteinases [23]. Possible implications of these findings, e.g., for mechanical ventilation or pathogen invasion during ventilator-associated pneumonia, have been highlighted [24].

Given these recent data and evidence from studies on the endothelial glycocalyx, it is obvious that the alveolar epithelial glycocalyx should have important functions in lung health and, when altered, in lung disease. These may include the regulation of alveolar fluid balance, the permeability of the air-blood barrier and the binding of inhaled pathogens. Based on the water binding capacity of glycocasaminoglycans like hyaluronan (where one molecule can bind up to 6000 molecules of water [25]), the alveolar epithelial glycocalyx must be involved in the regulation of the thickness and viscosity of the aqueous hypophase of the alveolar lining layer. This smooth lining layer has been estimated in rat lungs to have an area-weighted average thickness of 200 nm, with averages of 140 nm over flat parts of alveolar walls, 90 nm at alveolar junctions covering protrusions like capillaries and cell perikarya and 890 nm at alveolar junctions filling surface irregularities [8]. Using the area-weighted thickness of 200 nm and an alveolar surface area of 120 m^2^ in the human lung, one can calculate the volume of the alveolar lining layer to be 24 mL or about 1/3 mL per kg body weight. These values are in very good agreement with other estimates (summarized in [26]). Since the thickness of this alveolar lining layer contributes to the effective thickness of the air-blood barrier, which has to be crossed by oxygen diffusion, it directly determines the efficiency of gas exchange. Moreover, it is this alveolar lining layer where surfactant functions as anti-atelectatic agent.

## 3. Interactions Between Surfactant and the Glycocalyx

**Figure 1:**
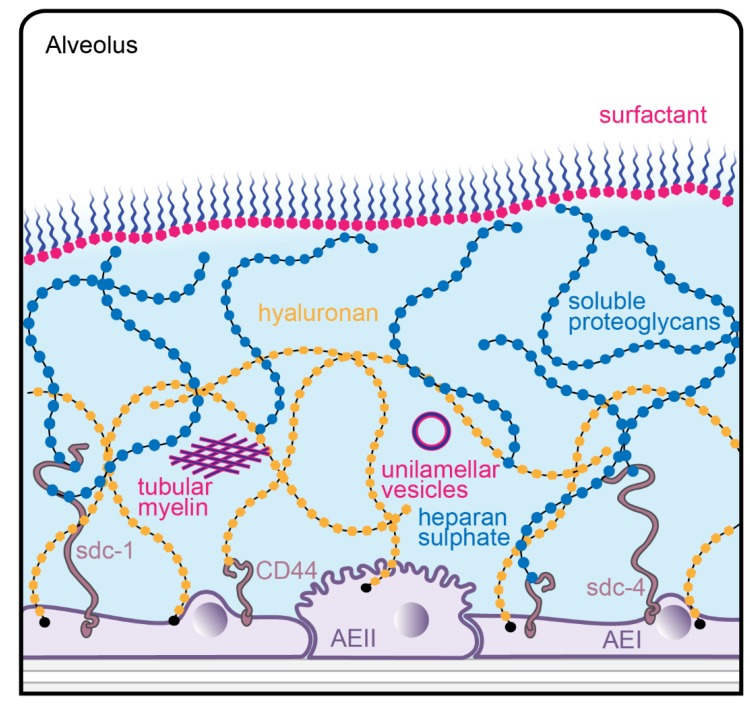
Schematic diagram of the alveolar lining layer with surfactant and glycocalyx components. Surfactant is present at the surface film and in the hypophase. Surfactant subtypes in the hypophase correspond to different stages in metabolism, shown here as active tubular myelin and inactive unilamellar vesicles. Proteoglycans of the glycocalyx can be attached to the apical membrane of type I (AEI) or type II (AEII) alveolar epithelial cells by syndecans like syndecan-1 (sdc-1) or syndecan-4 (sdc-4). Hyaluronan can bind either to its receptor CD44 or intercalates throughout the glyxcocalyx. The alveolar lining layer and its contents are not drawn to scale.

Compared to other surfaces in the body, a unique feature of the alveolar epithelial surface are the potential interactions of the glycocalyx with components of the surfactant system. However, these interactions are so far basically unexplored although a direct topographical relationship is obvious (Figure 1). One main component of the glycocalyx, hyaluronan, known to be secreted by AEII cells in vitro [27,28], has been shown to improve the biophysical (surface tension reducing) activity of surfactant in vitro and in vivo [29,30,31,32]. Thus, hyaluronan may have therapeutic potential in lung diseases where surfactant inactivation is a major pathophysiological event. One example of particular interest is the sequence from acute lung injury to fibrosis where alterations of alveolar micromechanics (alveolar instability and collapse induration) due to surfactant dysfunction have been demonstrated [33,34,35](for review, see [36,37]). Moreover, hyaluronan can act as an important signal for AEII cell renewal and repair. Accordingly, in patients with severe pulmonary fibrosis, hyaluronan on the cell surface of AEII cells is reduced [38].

Based on these findings, one may speculate on the potential relevance of the alveolar epithelial glycocalyx for surfactant function in general, and vice versa. Glycocalyx components may not only influence the resistance of surfactant against inactivation in lung injury, but may also be involved in the regulation of surfactant homeostasis and in the maintenance of the micro-environment in which surfactant acts (e.g., by regulating the thickness and viscosity of the hypophase of the alveolar lining layer) under normal conditions.

The surfactant proteins, being “the smart molecules in the surfactant system” [39], deserve particular attention in this context. The hydrophilic environment created by the glycocalyx may facilitate interactions with the hydrophilic surfactant proteins. Thus, SP-A and SP-D are attractive candidates for interactions with the glycocalyx. Their known immunomodulatory functions include the binding and opsonization of pathogens, interactions with cells of the innate and adaptive immune system and direct antimicrobial activity. As lung collectins, their basic structural units are homo-trimers which are formed via the triple helix of their collagen domain. The monomers consist of four sub-units: an N-terminal domain, a collagen domain, an α-helical neck domain, and a C-terminal C-type lectin domain which is often referred to as the carbohydrate recognition domain (CRD). These homo-trimers usually multimerize to higher-order oligomers. SP-A forms octadecamers (6 × 3) arranged as a bouquet of flowers while SP-D forms dodecamers (4 × 3) where the trimers connect via their N-terminal domains to form a cruciform structure. This means that both SP-A and SP-D can bind sugars with their CRD, with higher affinity for clustered oligosaccharides than for single monosaccharides and with both common and distinct saccharide-binding activities (for review, see [40,41,42,43,44]). Thus, it seems plausible that SP-A and SP-D may interact with sugars of the glycocalyx, and that this interaction will influence their functional capacity.

The hydrophobic SP-B and SP-C (reviewed in [45,46]) may also have their role in this scenario. Because hyaluronan is known to interact with phospholipids and has hydrophobic regions which could bind to SP-B and SP-C, the hypothesis that hyaluronan interacts with surfactant components to form a viscous gel in the hypophase of the alveolar lining layer has been put forward [47]. By this, hyaluronan would contribute to smoothing alveolar epithelial surface irregularities (e.g., folds or cell projections) via locally varying the thickness of the hypophase, thereby resulting in a smoothly curved air-liquid interface—a phenomenon well described for the alveolar lining layer [8,48,49].

Another cell type of relevance in this context are alveolar macrophages. They reside as free cells in the aqueous hypophase of the alveolar lining layer where they exert their phagocytic activity. One of their major functions is the removal and degradation of spent intraalveolar surfactant material. Alveolar macrophages express the hyaluronan receptor CD44 and thus are coated with bound hyaluronan promoting their survival [50]. Moreover, alveolar macrophages lacking CD44 have impaired lipid homeostasis leading to an increase in intraalveolar surfactant lipids [51]. This indicates an important role for hyaluronan in surfactant catabolism. 

Taken together, the coordinated interplay between surfactant and the glycocalyx is probably essential for alveolar micromechanics and lung function and thus deserves further attention.

## 4. Visualizing the Glycocalyx by Electron Microscopy

In order to gain a deeper understanding of the alveolar epithelial glycocalyx, one has to visualize it. The detailed architecture of the alveolar epithelium can only be resolved by EM (reviewed in [52]). Moreover, the high resolution offered by EM is also necessary to visualize the glycocalyx in its fine structural cell and tissue context. However, one has to bear in mind that no method of fixation for EM yields a “real in vivo” representation of lung structure. Although conventional chemical fixation for EM (based on glutaraldehyde and osmium tetroxide) results in good overall preservation of cell and tissue ultrastructure, the selective nature of the chemical interactions taking place during fixation, processing and dehydration (which are not very well understood) may induce artifacts. Therefore, morphological characteristics and differences observed in biological EM samples need to be interpreted with caution and knowledge of the preparation steps involved. This point is well appreciated in EM studies of lung surfactant (reviewed in [10,53]), but is at least as important for studies on the glycocalyx. 

Theoretically, the method of choice for near in vivo preservation of the glycocalyx by EM is avoiding chemical fixatives and applying cryo-EM of vitreous sections (CEMOVIS) [54,55]. The cooling conditions necessary to avoid cryo-fixation artifacts in EM (in particular ice crystal formation) are achieved by freezing very small tissue samples (thickness only up to 200 μm, i.e., roughly the diameter of a single alveolus in the human lung) with very high pressure (around 2000 bar) [56]. CEMOVIS can be used in selected singular experiments, e.g., to visualize surfactant-containing lamellar bodies within AEII cells [57]. Cryo-methods have occasionally also been used to visualize the glycocalyx, e.g., the combination of slam freezing and freeze substitution of bovine aorta and rat fat pad endothelial cells in vitro [58]. However, high pressure freezing leads to forced collapse of alveoli and thus destruction of the delicate alveolar lining layer where both the alveolar epithelial glycocalyx and the intraalveolar surfactant film are located. Moreover, this approach is not suitable for quantitative microscopic analyses of whole lungs by stereology because it precludes adequate sampling for which the whole (fixed) organ should be available [59]. At current, this can only be achieved by chemical fixation of the whole lung under carefully controlled conditions, either by airway instillation or, preferably, by vascular perfusion, both of which also fulfil the criteria of consistent reproducibility in space (homogeneity) and time (repeatability) [60]. Thus, at least for the near future, chemical fixation approaches will remain the routine method(s) of choice to study the alveolar epithelial glycocalyx in large-scale experimental studies.

Classical early EM studies on the surface coat of cells (for organs other than the lung, see e.g., [61,62,63]) have since then been refined by various fixation agents and protocols. With respect to the EM visualization of the glycocalyx, routine chemical fixation protocols usually yield poor results. Thus, the addition of staining agents that bind to components of the glycocalyx is necessary. These agents, usually of high atomic number, provide an “electron-dense” dark contrast because they scatter beam electrons [64]. These cytochemical methods work fine as structural tools. However, a molecular interpretation is problematic because of a severely limited specificity of the staining agents. Moreover, based on studies on the endothelial glycocalyx (reviewed in [15,17]), it is highly likely that all of these methods underestimate the real height of the glycocalyx to some degree. Nevertheless, the actual height of the alveolar lining layer (see above) also has to be taken into account. Therefore, caution is necessary when transferring findings from the endothelial glycocalyx, which is facing a liquid lumen, to the completely different physiological situation in the alveolar space where there is only a very thin liquid alveolar lining layer.

Among the stains that have been used for the glycocalyx are ruthenium red, colloidal iron, phosphotungstic acid, lanthanum nitrate, alcian blue and lectins such as concanavalin A, wheat germ agglutinin, and peanut agglutinin [64]. Several of these methods have also been applied to the alveolar surface, e.g., colloidal iron [65,66], ruthenium red [67,68], phosphotungstic acid [69], and concanavalin A [68,70,71]. In some of them, a differential staining pattern between AEI and AEII cells was emphasized [66,69,70]. Lectin binding patterns for AEI and AEII cells have later been used as surface markers for these cell types (e.g., [72,73,74]), although results were not always consistent. These early studies have been nicely reviewed by Martins and Bairos [75]. 

**Figure 2:**
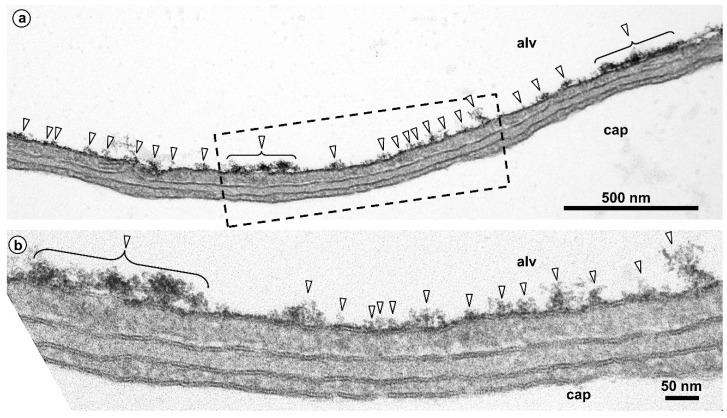
Mouse lung. Glycocalyx staining shown at lower (**a**) and higher (**b**) magnification. Alveolar lumen (alv) and capillary lumen (cap) are separated by the air-blood barrier consisting of a continuous alveolar epithelium, an interstitium and a continuous capillary endothelium. Here, a thin portion of the air-blood barrier is shown. The epithelium is made of thin extensions of alveolar epithelial type I cells. The interstitium is minimized to a common basal lamina shared by epithelium and endothelium. The endothelium is of the non-fenestrated type. The alveolar epithelial surface is clearly stained after treatment with colloidal thorium dioxide (arrowheads). The boxed area in (**a**) is shown in (**b**) at higher magnification.

**Figure 3:**
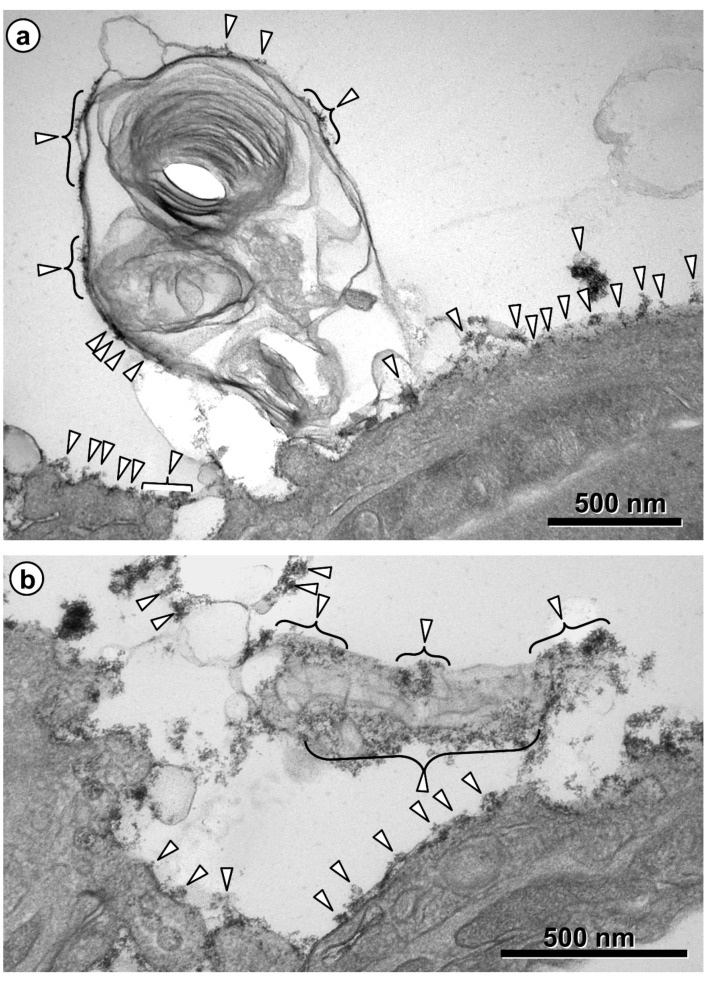
Mouse lung. Glycocalyx staining on intraalveolar surfactant. Intraalveolar surfactant material is visible on top of the alveolar epithelium as secreted lamellar body content (**a**) and as tubular myelin (**b**) with lattice-like structure. Both are stained, particularly at the outside, with thorium dioxide, like the surface of the alveolar epithelium (arrowheads).

**Figure 4:**
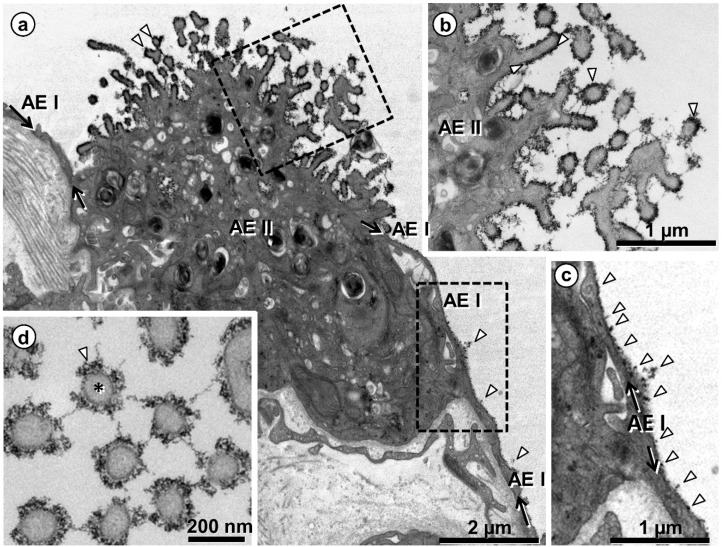
Human lung. Visualization of the glycocalyx on the surface of alveolar epithelial type I (AEI) and type II (AEII) cells. (**a**) Overview showing one AEII cell and neighboring thin AEI cell extensions, the lineage of the latter highlighted by arrows. The black lining and dots on the cell surfaces (arrowheads in (**a**)) depict the glycocalyx, marked by colloidal thorium dioxide. The boxed areas are shown in (**b**,**c**) at higher magnification. Note heavily stained microvilli (arrowheads in (**b**)) and staining at the apical cell membrane of AEI cell (arrowheads in (**c**)). (**d**) Profiles of cross-sectioned microvilli (one of them marked by asterisk) of an AEII cell. The glycocalyx (arrowhead in (**d**)) surrounds the microvilli and also appears as threads between them.

**Figure 5:**
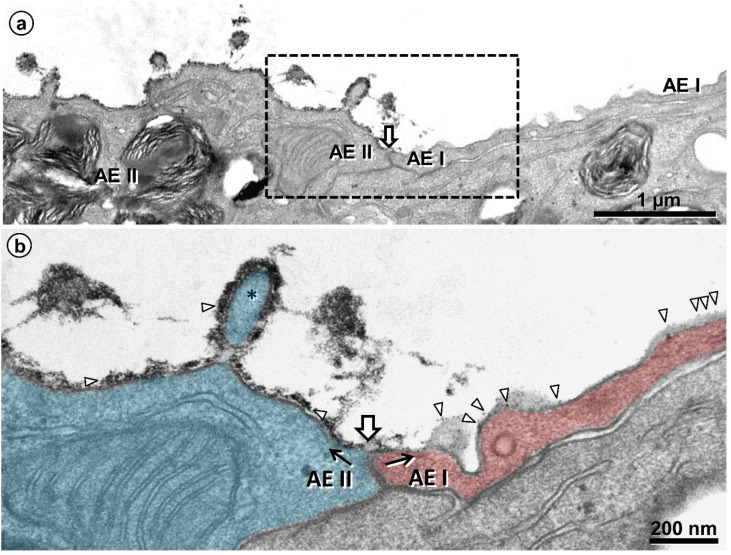
Human lung. Comparison of the glycocalyx of an alveolar epithelial type I (AEI) and type II (AEII) cell. (**a**) Alveolar surface depicted in sequence from an AEII cell (left) across the cell contact (block arrow) to an AEI cell (right). The boxed area is shown in (**b**) at higher magnification. (**b**) Different intensity of glycocalyx on the AEII (left, colored blue) and AEI (right, colored red) cell (along black arrows). The thorium dioxide deposition (marked by arrowheads) is intense on the AEII cell, forming a nearly continuous layer also visible on a microvillus (asterisk), while it appears rather punctual on the AEI cell.

**Figure 6:**
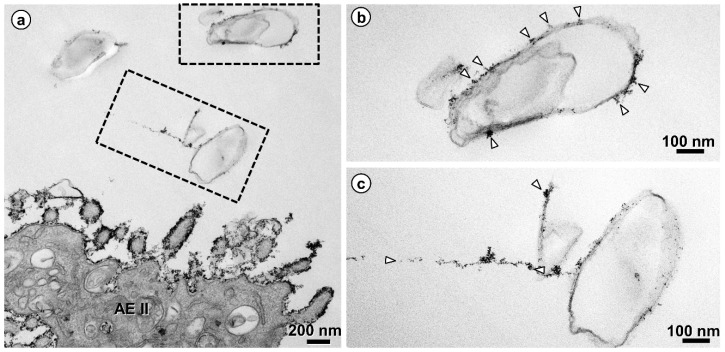
Human lung. Glycocalyx staining on intraalveolar surfactant. (**a**) Intraalveolar surfactant (boxed areas) in the airspace of an alveolus nearby an alveolar epithelial type II (AEII) cell is decorated by thorium dioxide. The boxed areas are shown in (**b**,**c**) at higher magnification. The stain on the intraalveolar surfactant (arrowheads) is less intense compared to the stain on the AEII cell, rather comparable with the punctual stain on an AEI cell (compare Figure 4).

One staining method of particular interest for the EM visualization of the glycocalyx is based on cationic hydrous thorium dioxide colloids (cThO_2_), which has been shown to allow for better tissue penetration and more intense staining compared to other staining agents [76,77]. The size of the cThO_2_ particles ranges from 1 to 1.7 nm. This approach has been applied successfully to study the glomerular endothelial glycocalyx in the kidney [78]. We have transferred this method to the lung in order to investigate the ultrastructure of the alveolar epithelial glycocalyx and potential associations with surfactant. Fixed samples of 1 to 2 mm edge length from mouse and human lungs were immersed in 100 mM sodium acetate buffer (NaAc) at pH 3.0 for 5 min, in 0.5% cThO_2_ in NaAc for 5 min and again in NaAc for 5 min, followed by further post-fixation and embedding according to our routine lung EM protocol [79]. During immersion in NaAc and cThO_2_, samples were gently massaged with a wooden skewer to allow the solutions to diffuse into the alveoli. The low pH increases the specificity for negatively charged glycocalyx components. Results based on this protocol are shown and described for mouse (Figure 2 and Figure 3) and human (Figure 4, Figure 5 and Figure 6) lung. 

Taken together, these findings indicate: (1) differences in the structure of the glycocalyx between the two cell types of the alveolar epithelium, with AEII cells showing a stronger staining, thus confirming earlier studies [66,69,70]; and (2) direct interactions of the alveolar epithelial glycocalyx with intraalveolar surfactant subtypes, including tubular myelin. These findings have several implications. In contrast to AEI cells, AEII cells possess numerous microvilli. Besides their function of providing an increased apical cell surface area, it is obvious that they are also effective as glycocalyx carriers. Interconnections between microvilli strongly contribute to the three-dimensional meshwork arrangement of the alveolar epithelial glycocalyx. Moreover, one has to take the topographical distribution of the two alveolar epithelial cell types into consideration. AEI cells, which cover about 95% of the alveolar surface, serve their lining function by providing thin cell extensions to the flat parts of the air-blood barrier that are optimized for efficient diffusion. On the other hand, the cuboidal AEII cells exert their secretory and renewal function mainly in the corners of alveoli where alveolar junctions are located. Thus, the well-known regional differences in the height of the alveolar lining layer between flat parts and junctions of alveoli (see above; [8]) are likely reflected in regional differences in the alveolar epithelial glycocalyx. Regarding the close association of the alveolar epithelial glycocalyx with intraalveolar surfactant, it is noteworthy that a similar staining pattern (i.e., at the outer lamellae of freshly secreted lamellar bodies and tubular myelin) has been shown by immunoelectron microscopy for SP-A in the human lung [80]. These morphological findings consolidate the notion of direct interactions of SP-A with components of the alveolar epithelial glycocalyx. This may be of particular relevance for tubular myelin, a still incompletely understood highly-ordered intraalveolar surfactant subtype. The characteristic lattice-like structure of tubular myelin requires the presence of SP-A which is located in the lattice with its CRD directed towards the corners [42,44,81], but an association of tubular myelin with carbohydrates has also been described [68,71]. Assumed to be an intermediate precursor of the surface film, tubular myelin may actually serve a dual function: not only as intraalveolar reservoir of surfactant lipids, but also as a “spider’s web” for alveolar host defence that could be supported by the glycocalyx.

## 5. Conclusions and Outlook

It is remarkable that early studies on AEII cells, the surfactant system and the alveolar lining layer (even before these now common names were coined) already suggested the presence of carbohydrates in this context. In 1954, Charles Clifford Macklin not only predicted the presence of an “alveolar mucoid film”, but also estimated (“arbitrarily”) its thickness to be 200 nm (confirmed more than 40 years later [8]) and suggested its components to be “acid mucopolysaccharides and myelinogens”, i.e., glycosaminoglycans and phospholipids [82]. After the actual discovery of surfactant by John Clements [83], Bolande and Klaus, based on light microscopic findings, suggested that, besides phospholipids, “a mucopolysaccharide fraction may also be present” in the alveolar lining layer [84]. Similarly, Groniowski and Biczyskowa studied the alveolar lining layer by EM and found evidence indicating “the existence of another component of the acidic mucopolysaccharide nature” [65]. In that sense, we have to reintroduce the glycocalyx into our concepts of the alveolar lining layer and the surfactant system.

The spatial interaction between surfactant and the glycocalyx at the alveolar epithelial surface may generate a “win-win” situation, where surfactant benefits from glycocalyx components such as hyaluronan, warranting its biophysical function. Conversely, surfactant may contribute to the integrity and function of the alveolar epithelial glycocalyx. It is unknown how the content of the alveolar hypophase is sensed, e.g., with respect to its volume, viscosity and surfactant pool size. This remains to be investigated both under normal and challenge conditions such as acute lung injury and pulmonary infection. Integrating the glycocalyx into our picture of the lung alveolus also offers attractive therapeutic perspectives, e.g., by adding glycocalyx components to exogenous surfactant preparations. Moreover, engineering of synthetic glycocalyx [85] provides a very promising approach for the preservation and/or reconstitution of the unique micro-environment on top of the alveolar surface. New developments in EM should be included in such studies, e.g., volume correlative light and electron microscopy techniques [86,87]. It seems timely to explore the structure and function of the alveolar epithelial glycocalyx, in particular its relations to the surfactant system. Given its potential importance, it should no longer remain an enigma.

## References

[1] Schneider J.P., Wrede C., Hegermann J., Weibel E.R., Mühlfeld C., Ochs M. (2019). On the topological complexity of human alveolar epithelial type 1 cells. Am. J. Respir. Crit. Care Med..

[2] Ochs M., Weibel E.R., Grippi M.A., Elias J.A., Fishman J.A., Kotloff R.M., Pack A.I., Senior R.M. (2015). Functional design of the human lung for gas exchange. Fishman´s Pulmonary Diseases and Disorders.

[3] Hsia C.C.W., Hyde D.M., Weibel E.R. (2016). Lung structure and the intrinsic challenges of gas exchange. Compr. Physiol..

[4] Low F.N. (1952). Electron microscopy of the rat lung. Anat. Rec..

[5] Low F.N. (1953). The pulmonary alveolar epithelium of laboratory animals and man. Anat. Rec..

[6] Weibel E.R., Gil J. (1968). Electron microscopic demonstration of an extracellular duplex lining layer of alveoli. Respir. Physiol..

[7] Gil J., Weibel E.R. (1969). Improvements in demonstration of lining layer of lung alveoli by electron microscopy. Respir. Physiol..

[8] Bastacky J., Lee C.Y.C., Goerke J., Koushafar H., Yager D., Kenaga L., Speed T.P., Chen Y., Clements J.A. (1995). Alveolar lining layer is thin and continuous: Low-temperature scanning electron microscopy of rat lung. J. Appl. Physiol..

[9] Perez-Gil J. (2008). Structure of pulmonary surfactant membranes and films: The role of proteins and lipid-protein interactions. Biochim. Biophys. Acta.

[10] Ochs M. (2010). The closer we look the more we see? Quantitative microscopic analysis of the pulmonary surfactant system. Cell. Physiol. Biochem..

[11] Orgeig S., Morrison J.L., Daniels C.B. (2016). Evolution, development and function of the pulmonary surfactant system in normal and perturbed environments. Compr. Physiol..

[12] Olmeda B., Martinez-Calle M., Perez-Gil J. (2017). Pulmonary surfactant metabolism in the alveolar airspace: Biogenesis, extracellular conversions, recycling. Ann. Anat..

[13] Knudsen L., Ochs M. (2018). The micromechanics of lung alveoli: Structure and function of surfactant and tissue components. Histochem. Cell Biol..

[14] Bennett H.S. (1963). Morphological aspects of extracellular polysaccharides. J. Histochem. Cytochem..

[15] Reitsma S., Slaaf D.W., Vink H., van Zandvoort M.A.M.J., oude Egbrink M.G.A. (2007). The endothelial glycocalyx: Composition, functions, and visualization. Pfluegers Arch..

[16] Tarbell J.M., Cancel L.M. (2016). The glycocalyx and its significance in human medicine. J. Intern. Med..

[17] LaRiviere W.B., Schmidt E.P. (2018). The pulmonary endothelial glycocalyx in ARDS: A critical role of heparan sulphate. Curr. Top. Membr..

[18] Schmidt E.P., Yang Y., Janssen W.J., Gandjeva A., Perez M.J., Barthel L., Zemans R.L., Bowman J.C., Konayagi D.E., Yunt Z.X. (2012). The pulmonary endothelial glycocalyx regulates neutrophil adhesion and lung injury during experimental sepsis. Nat. Med..

[19] Inagawa R., Okada H., Takemura G., Suzuki K., Takada C., Yano H., Ando Y., Usui T., Hotta Y., Miyazaki N. (2018). Ultrastructural alteration of pulmonary capillary endothelial glycocalyx during endotoxemia. Chest.

[20] Biasin V., Wygrecka M., Bärnthaler T., Jandl K., Jain P.P., Balint Z., Kovacs G., Leitinger G., Kolb-Lenz D., Kornmueller K. (2018). Docking of meprin α to heparan sulphate protects the endothelium from inflammatory cell extravasation. Thromb. Haemost..

[21] Li Q., Park P.W., Wilson C.L., Parks W.C. (2002). Matrilysin shedding of syndecan-1 regulates chemokine mobilization and transepithelial efflux of neutrophils in acute lung injury. Cell.

[22] Pruessmeyer J., Martin C., Hess F.M., Schwarz N., Schmidt S., Kogel T., Hoettecke N., Schmidt B., Secht A., Uhlig S. (2010). A disintegrin and metalloproteinase 17 (ADAM17) mediates inflammation-induced shedding of syndecan-1 and -4 by lung epithelial cells. J. Biol. Chem..

[23] Haeger S.M., Liu X., Han X., McNeil J.B., Oshima K., McMurty S.A., Yang Y., Ouyang Y., Zhang F., Nozik-Grayck E. (2018). Epithelial heparan sulfate contributes to alveolar barrier function and is shed during lung injury. Am. J. Respir. Cell Mol. Biol..

[24] Weidenfeld S., Kuebler W.M. (2018). Shedding first light on the alveolar epithelial glycocalyx. Am. J. Respir. Cell Mol. Biol..

[25] Allegra L., Patrona S.D., Petrigni G. (2012). Hyaluronic acid: Perspectives in lung disease. Handb. Exp. Pharmacol..

[26] Walters D.V. (2002). Lung lining liquid—The hidden depths. Biol. Neonate.

[27] Sahu S.C., Tanswell A.K., Lynn W.S. (1980). Isolation and characterization of glycosaminoglycans secreted by human foetal lung type II pneumocytes in culture. J. Cell Sci..

[28] Skinner S.J.M., Post M., Torday J.S., Stiles A.D., Smith B.T. (1987). Characterization of proteoglycans synthesized by fetal rat lung type II pneumonocytes in vitro and the effects of cortisol. Exp. Lung Res..

[29] Lu K.W., Goerke J., Clements J.A., Taeusch H.W. (2005). Hyaluronan decreases surfactant inactivation in vitro. Pediatr. Res..

[30] Taeusch H.W., de la Bernardino Serra J., Perez-Gil J., Alonso C., Zasadzinski J.A. (2005). Inactivation of pulmonary surfactant due to serum-inhibited adsorption and reversal by hydrophilic polymers: Experimental. Biophys. J..

[31] Wang X., Sun Z., Qian L., Guo C., Yu W., Wang W., Lu K.W., Taeusch H.W., Sun B. (2006). Effects of hyaluronan-fortified surfactant in ventilated premature piglets with respiratory distress. Biol. Neonate.

[32] Lopez-Rodriguez E., Cruz A., Richter R., Taeusch H.W., Perez-Gil J. (2013). Transient exposure of pulmonary surfactant to hyaluronan promotes structural and compositional transformations into a highly active state. J. Biol. Chem..

[33] Lutz D., Gazdhar A., Lopez-Rodriguez E., Ruppert C., Mahavadi P., Günther A., Klepetko W., Bates J.H., Smith B., Geiser T. (2015). Alveolar derecruitment and collapse induration as crucial mechanisms in lung injury and fibrosis. Am. J. Respir. Cell Mol. Biol..

[34] Steffen L., Ruppert C., Hoymann H.G., Funke M., Ebener S., Kloth C., Mühlfeld C., Ochs M., Knudsen L., Lopez-Rodriguez E. (2017). Surfactant replacement therapy reduces acute lung injury and collapse induration related lung remodeling in the bleomycin model. Am. J. Physiol. Lung Cell. Mol. Physiol..

[35] Zhou T., Yu Z., Jian M.Y., Ahmad I., Trempus C., Wagener B.M., Pittet J.F., Aggarwal S., Garantziotis S., Song W. (2018). Instillation of hyaluronan reverses acid instillation injury to the mammalian blood gas barrier. Am. J. Physiol. Lung Cell. Mol. Physiol..

[36] Todd N.W., Atamas S.P., Luzina I.G., Galvin J.R. (2015). Permanent alveolar collapse is the predominant mechanism in idiopathic pulmonary fibrosis. Expert Rev. Respir. Med..

[37] Knudsen L., Ruppert C., Ochs M. (2017). Tissue remodelling in pulmonary fibrosis. Cell Tissue Res..

[38] Liang J., Zhang Y., Xie T., Liu N., Chen H., Geng Y., Kurkciyan A., Stripp B.R., Jiang D., Noble P.W. (2016). Hyaluronan and TLR4 promote surfactant-protein-C-positive alveolar progenitor cell renewal and prevent severe pulmonary fibrosis. Nat. Med..

[39] Hawgood S., Clements J. (1990). Pulmonary surfactant and its apoproteins. J. Clin. Investig..

[40] Hawgood S., Poulain F.R. (2001). The pulmonary collectins and surfactant metabolism. Annu. Rev. Physiol..

[41] Crouch E., Wright J.R. (2001). Surfactant proteins A and D and pulmonary host defense. Annu. Rev. Physiol..

[42] McCormack F.X., Whitsett J.A. (2002). The pulmonary collectins, SP-A and SP-D, orchestrate innate immunity in the lung. J. Clin. Investig..

[43] Wright J.R. (2005). Immunoregulatory functions of surfactant proteins. Nat. Rev. Immunol..

[44] Kingma P.S., Whitsett J.A. (2006). In defense of the lung: Surfactant protein A and surfactant protein B. Curr. Opin. Pharmacol..

[45] Weaver T.E., Conkright J.J. (2001). Functions of surfactant proteins B and C. Annu. Rev. Physiol..

[46] Whitsett J.A., Weaver T.E. (2002). Hydrophobic surfactant proteins in lung function and disease. N. Engl. J. Med..

[47] Bray B.A. (2001). The role of hyaluronan in the pulmonary alveolus. J. Theor. Biol..

[48] Weibel E.R., Gil J., West J.B. (1977). Structure-Function Relationships at the Alveolar Level. Bioengineering Aspects of the Lung.

[49] Rühl N., Lopez-Rodriguez E., Albert K., Smith B.J., Waever T.E., Ochs M., Knudsen L. (2019). Surfactant protein B deficiency induced high surface tension: Relationship between alveolar micromechanics, alveolar fluid properties and alveolar epithelial cell injury. Int. J. Mol. Sci..

[50] Dong Y., Poon G.F.T., Arif A.A., Lee-Sayer S.S.M., Dosanjh M., Johnson P. (2018). The survival of fetal and bone-marrow monocyte-derived alveolar macrophages is promoted by CD44 and its interaction with hyaluronan. Mucosal Immunol..

[51] Dong Y., Arif A.A., Guo J., Ha Z., Lee-Sayer S.S.M., Poon G.F.T., Dosanjh M., Roskelley C.D., Huan T., Johnson P. (2020). CD44 loss disrupts lung lipid surfactant homeostasis and exacerbates oxidized lipid-induced lung inflammation. Front. Immunol..

[52] Ochs M., Knudsen L., Hegermann J., Wrede C., Grothausmann R., Mühlfeld C. (2016). Using electron microscopes to look into the lung. Histochem. Cell Biol..

[53] Gil J. (1985). Histological preservation and ultrastructure of alveolar surfactant. Annu. Rev. Physiol..

[54] Al-Amoudi A., Chang J.J., Leforestier A., McDowall A., Salamin L.M., Norlen L.P.O., Richter K., Blanc N.S., Studer D., Dubochet J. (2004). Cryo-electron microscopy of vitreous sections. EMBO J..

[55] Dubochet J. (2011). Cryo-EM—The first 30 years. J. Microsc..

[56] Studer D., Humbel B., Chiquet M. (2008). Electron microscopy of high pressure frozen samples: Bridging the gap between cellular ultrastructure and atomic resolution. Histochem. Cell Biol..

[57] Vanhecke D., Herrmann G., Graber W., Hillmann-Marti T., Mühlfeld C., Studer D., Ochs M. (2010). Lamellar body ultrastructure revisited: High-pressure freezing and cryo-electron microscopy of vitreous sections. Histochem. Cell Biol..

[58] Ebong E.E., Macaluso F.P., Spray D.C., Tarbell J.M. (2011). Imaging the endothelial glycocalyx in vitro by rapid freezing/freeze substitution transmission electron microscopy. Arterioscler. Thromb. Vasc. Biol..

[59] Hsia C.C.W., Hyde D.M., Ochs M., Weibel E.R. (2010). An official research policy statement of the American Thoracic Society / European Respiratory Society: Standards for quantitative assessment of lung structure. Am. J. Respir. Crit. Care Med..

[60] Weibel E.R., Otis A.B. (1984). Morphometric and stereological methods in respiratory physiology, including fixation techniques. Techniques in the Life Sciences. Techniques in Respiratory Physiology.

[61] Ito S. (1956). The enteric surface coat on cat intestinal microvilli. J. Cell Biol..

[62] Luft J.H. (1964). Electron microscopy of cell extraneous coats as revealed by ruthenium red staining. J. Cell Biol..

[63] Rambourg A., Leblond C.P. (1967). Electron microscope observations on the carbohydrate-rich cell coat present at the surface of cells in the rat. J. Cell Biol..

[64] Hayat M.A. (1993). Stains and Cytochemical Methods.

[65] Groniowski J., Biczyskowa W. (1964). Structure of the alveolar lining film of the lungs. Nature.

[66] Kuhn C. (1968). Cytochemistry of pulmonary alveolar epithelial cells. Am. J. Pathol..

[67] Brooks R.E. (1969). Ruthenium red stainable surface layer on lung alveolar cells; electron microscopic interpretation. Stain Technol..

[68] Bignon J., Faubert F., Jaurand M.C. (1976). Plasma protein immunocytochemistry and polysaccharide cytochemistry at the surface of alveolar and endothelial cells in the rat lung. J. Histochem. Cytochem..

[69] Adamson I.Y.R., Bowden D.H. (1970). The surface complexes of the lung. Am. J. Pathol..

[70] Roth J. (1973). Ultrahistochemical demonstration of saccharide components of complex carbohydrates at the alveolar cell surface and at the mesothelial cell surface of the pleura visceralis of mice by means of concanavalin A. Exp. Pathol..

[71] Nir I., Pease D.C. (1976). Polysaccharides in lung alveoli. Am. J. Anat..

[72] Meban C. (1986). Ultrastructural visualisation of carbohydrate groups in the surface coating of hamster alveolar macrophages and pneumonocytes. J. Anat..

[73] Taatjes D.J., Barcomb L., Leslie K.O., Low R.B. (1990). Lectin binding patterns to terminal sugars of rat lung alveolar epithelial cells. J. Histochem. Cytochem..

[74] Iwatsuki H., Sasaki K., Suda M., Itano C. (1993). Cell differentiation of alveolar epithelium in the developing rat lung: Ultrahistochemical studies of glycoconjugates on the epithelial cell surface. Histochemistry.

[75] Martins M.F., Bairos V.A. (2002). Glycocalyx of lung epithelial cells. Int. Rev. Cytol..

[76] Groot C.G. (1981). Positive colloidal thorium dioxide as an electron microscopical contrasting agent for glycosaminoglycans, compared with ruthenium red and positive colloidal iron. Histochemistry.

[77] Lünsdorf H., Kristen I., Barth E. (2006). Cationic hydrous thorium dioxide colloids—A useful tool for staining negatively charged surface matrices of bacteria for use in energy-filtered transmission electron microscopy. BMC Microbiol..

[78] Hegermann J., Lünsdorf H., Ochs M., Haller H. (2016). Visualization of the glomerular endothelial glycocalyx by electron microscopy using cationic colloidal thorium dioxide. Histochem. Cell Biol..

[79] Mühlfeld C., Rothen-Rutishauer B., Vanhecke D., Blank F., Gehr P., Ochs M. (2007). Visualization and quantitative analysis of nanoparticles in the respiratory tract by transmission electron microscopy. Part. Fibre Toxicol..

[80] Ochs M., Johnen G., Müller K.M., Wahlers T., Hawgood S., Richter J., Brasch F. (2002). Intracellular and intraalveolar localization of surfactant protein A (SP-A) in the parenchymal region of the human lung. Am. J. Respir. Cell Mol. Biol..

[81] Voorhout W.F., Veenendaal T., Haagsman H.P., Verkleij A.J., Van Golde L.M.G., Geuze H.J. (1991). Surfactant protein A is localized at the corners of the pulmonary tubular myelin lattice. J. Histochem. Cytochem..

[82] Macklin C.C. (1954). The pulmonary alveolar mucoid film and the pneumonocytes. Lancet.

[83] Clements J.A. (1957). Surface tension of lung extracts. Proc. Soc. Exp. Biol. Med..

[84] Bolande R.P., Klaus M.H. (1964). The morphologic demonstration of an alveolar lining layer and its relationship to pulmonary surfactant. Am. J. Pathol..

[85] Purcell S.C., Godula K. (2019). Synthetic glycoscapes: Addressing the structural and functional complexity of the glycocalyx. Interface Focus.

[86] Fang T., Lu X., Berger D., Gmeiner C., Cho J., Schalek R., Ploegh H., Lichtman J. (2018). Nanobody immunostaining for correlated light and electron microscopy with preservation of ultrastructure. Nat. Methods.

[87] Hegermann J., Wrede C., Fassbender S., Schliep R., Ochs M., Knudsen L., Mühlfeld C. (2019). Volume-CLEM: A method for correlative light and electron microscopy in three dimensions. Am. J. Physiol. Lung Cell. Mol. Physiol..

